# Fretting Fatigue Behaviour of Pin-Loaded Thermoset Carbon-Fibre-Reinforced Polymer (CFRP) Straps

**DOI:** 10.3390/polym8040124

**Published:** 2016-04-07

**Authors:** Fabio Baschnagel, Vanessa Rohr, Giovanni Pietro Terrasi

**Affiliations:** 1 Mechanical Systems Engineering Laboratory, Empa, Swiss Federal Laboratories for Materials Science and Technology, Überlandstrasse 129, CH-8600 Dübendorf, Switzerland; fabio.baschnagel@empa.ch; 2 Laboratory of Composite Materials and Adaptive Structures, ETH Zürich, Leonhardstrasse 27, CH-8092 Zürich, Switzerland; vrohr@student.ethz.ch

**Keywords:** carbon-fibre-reinforced polymer, CFRP, fretting, fatigue, FEA

## Abstract

This paper focuses on the fretting fatigue behaviour of pin-loaded carbon-fibre-reinforced polymer (CFRP) straps studied as models for rigging systems in sailing yachts, for suspenders of arch bridges and for pendent cables in cranes. Eight straps were subjected to an ultimate tensile strength test. In total, 26 straps were subjected to a fretting fatigue test, of which ten did not fail. An S–N curve was generated for a load ratio *R* of 0.1 and a frequency *f* of 10 Hz, showing a fatigue limit stress of the straps around the matrix fatigue limit, corresponding to 46% of the straps’ ultimate tensile strength (σ_UTS_). The fatigue limit was defined as 3 million load cycles (*N* = 3 × 10^6^), but tests were even conducted up to *N* = 11.09 × 10^6^. Catastrophic failure of the straps was initiated in their vertex areas. Investigations on the residual strength and stiffness properties of straps tested around the fatigue limit stress (for *N* ≥ 1 × 10^6^) showed little influence of the fatigue loading on these properties. Quasi-static finite element analyses (FEA) were conducted. The results obtained from the FEA are in good agreement with the experiments and demonstrate a fibre parallel stress concentration in the vertex area of factor 1.3, under the realistic assumption of a coefficient of friction (*cof*) between pin and strap of 0.5.

## 1. Introduction

**Figure 1:**
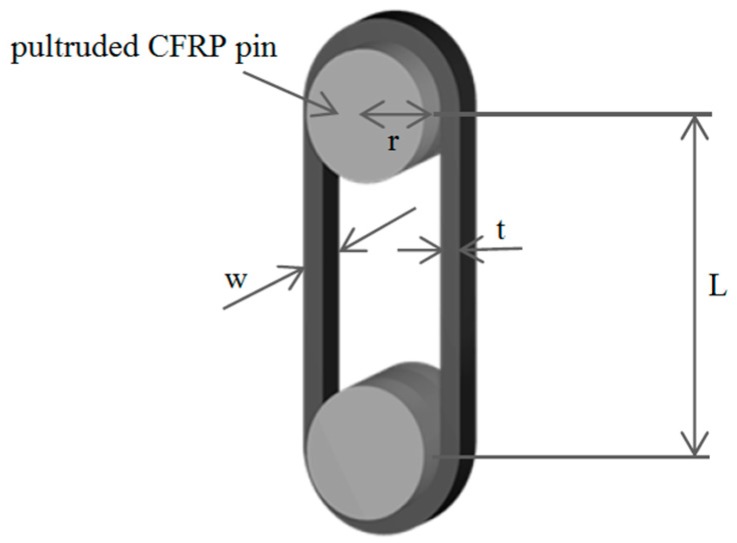
Schematic of a pin-loaded CFRP strap.

By weighing six times less than comparable steel riggings, rigging systems made from CFRP have recently become more and more important in the sailing and construction industry [1], where they are starting to replace steel ropes that are prone to environmental influences such as salt water, which can cause stress corrosion. However, the matrix system in CFRPs is known to deteriorate due to environmental influences such as UV light or suffer from static fatigue (e.g., creep). In applications such as rigging systems for sailing yachts or in suspenders used for half-through arch bridges [2], dynamic fatigue is highly relevant as well. It causes mechanical property degradation under cyclic loading that can lead to failure of the component. In the present study, the fretting, which is caused by the relative movement between the outer surface of the contacting pin and the inner surface of the strap (see Figure 1), further increases the material degradation [3,4,5]. Compared to isotropic materials such as metals, ceramics or polymers, the damage modes and crack propagation are much more complex in CFRPs and depend on a variety of factors such as the fibres, the matrix, the sizing (fibre-matrix interaction), the layup sequence of the laminate or the loading conditions, only to name a few [6,7,8,9]. Despite this vast amount of influencing parameters, Reifsnider [10] characterized the fatigue behaviour of multiaxial fibre reinforced composites (FRPs) in a general way and divided it into three stages. In the first stage, the damage develops at a very rapid rate within the first 10%–15% of the laminate’s life. In this stage, the major damage mode is matrix cracking in the laminae with the most off-axis fibre orientation. This intralaminar matrix cracking between the fibres reaches a uniform saturation spacing at the end of stage I, called characteristic damage state (CDS). In stage II, comprising 70%–80% of the fatigue life, damage is still initiated and the already existing damages continue to grow, but at a much slower rate, until the laminate is severely damaged and then enters stage III, where the damage process is accelerated again until final failure of the laminate.

In the case of purely unidirectional (UD) CFRP laminates, the situation is different, since no off-axis plies are present. However, since carbon fibres show excellent fatigue behaviour [11], the matrix and its interaction with the fibres remains the limiting factor in the composite’s fatigue life. This was shown in studies by P.T. Curtis [12,13,14], where the influence on the fatigue limit of different carbon fibres in the same matrix system (epoxy) or the same carbon fibre in different epoxy resins was investigated. Curtis showed that composites reached the same fatigue limit with the same epoxy resin and fibre volume content (*V*_f_), irrespective of the type of carbon fibre used as reinforcement. On the other hand, embedding the same fibre in different matrix systems resulted in varying fatigue limits. Based on the same idea, Talreja [15] introduced a fatigue life diagram in which he suggests that the fatigue life of a unidirectionally reinforced polymer is governed by the composite’s quasi-static fracture strain and the matrix’ fatigue limit strain. He argues that first, irreversible damage of the matrix as well as fibre breaking occur if a certain strain level is reached, irrespective of the *V*_f_ or stress level. With the matrix fatigue limit strain being at ε_mf_ = 0.6% for epoxy resins [16], no damage progression takes place in the composite below this strain level, given the carbon fibres’ insensitiveness to fatigue.

This assumption of course only holds if no fretting of the unidirectional CFRP laminate is present or if the fretting stops at some point. The negative influence of a fretting partner on the fatigue life of a CFRP composite was investigated in studies by Schulte *et al.* [3,4,5]. They could show how an increased normal contact force leads to a more pronounced decrease in fatigue strength and that the influence of the contact force increases if the contacting ply is oriented in the primary loading direction. This is also the case in the examined pin-loaded CFRP straps, and the goal of this study was to investigate the fretting fatigue behaviour of these straps in contact with a pultruded CFRP pin in order to improve the durability of existing CFRP riggings.

## 2. Materials and Manufacturing

**Figure 2:**
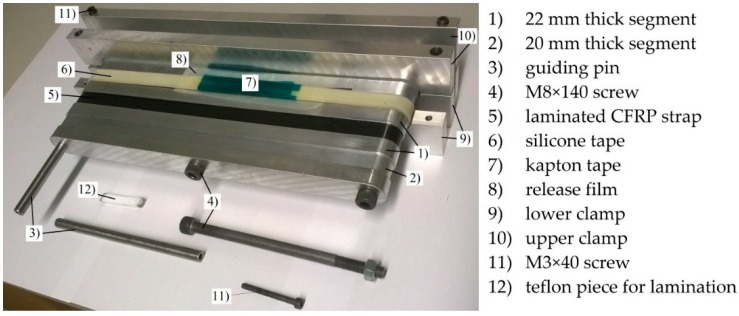
Mould for lamination of the CFRP straps.

The materials used for the production of the out of autoclave (OOA) carbon fibre/epoxy (CF-EP) prepreg were a XB3515/Aradur^®^ 5021 matrix system by Huntsman (Huntsman Advanced Materials GmbH, Basel, Switzerland) and intermediate modulus IMS60 carbon fibres by Toho Tenax^®^ (Toho Tenax Europe GmbH, Wuppertal, Germany) with a reported Young’s modulus of 290 GPa and tensile strength of 5,600 MPa. The 12 ± 1 mm and 9 ± 1 mm wide prepreg tapes were produced by Carbo-Link AG in Fehraltorf, Switzerland and reported to have a *V*_f_ of 62% ± 2% with an average measured areal weight of 271 g/m^2^. The laminate’s average experimental tensile strength was 2,567 ± 58 MPa with a longitudinal (fibre parallel) elastic modulus *E*_11_ of 168 ± 6.6 GPa and an average ultimate tensile strain ε_11u_ of 1.52% ± 0.23%. The experiments were conducted according to DIN EN 2561 [17] and five samples were tested. It is believed that the 1.9% failure strain claimed by the manufacturer was not reached due to the presence of wavy fibres. The presence of fibre misalignment was confirmed by investigating polished samples of the laminate’s cross section under an optical microscope. Further investigations on the laminate quality of the laminated straps revealed an average *V*_f_ of 66% (tested according to DIN EN 2564 [18]) and a void content of less than 1% (determined by image analysis of polished samples inspected under an optical microscope). The 50 mm long pins used for the loading of the straps were cut from 2 m long pultruded T300 carbon fibre reinforced epoxy rods with a *V*_f_ of 60%–65% [19]. The CFRP straps had a thickness *t* of 1 mm, a shaft length *L* of 250 mm, a width *w* of 12 mm or 9.7 mm, depending on the prepreg tape used, and an inner radius *r* of 10 mm. The schematic is shown in Figure 1. The straps were laminated by winding the prepreg tapes around an aluminium mould consisting of 11 segments that were screwed together as shown in Figure 2, with the thicker segments acting as lateral boundary for the prepreg tapes. The winding resulted in an overlap of the tape of approximately 150 mm in one shaft. A silicone tape was placed on top of the laminate to ensure a more homogeneous pressure distribution on the laminate during curing. Two clamps were placed on each side of the mould and screwed together in order to compress the laminate. The straps were cured at 140 °C for 2.5 h and conditioned for at least 48 h at 23 °C and 50% R.H. (relative humidity) before testing.

## 3. Experimental Setup

**Figure 3:**
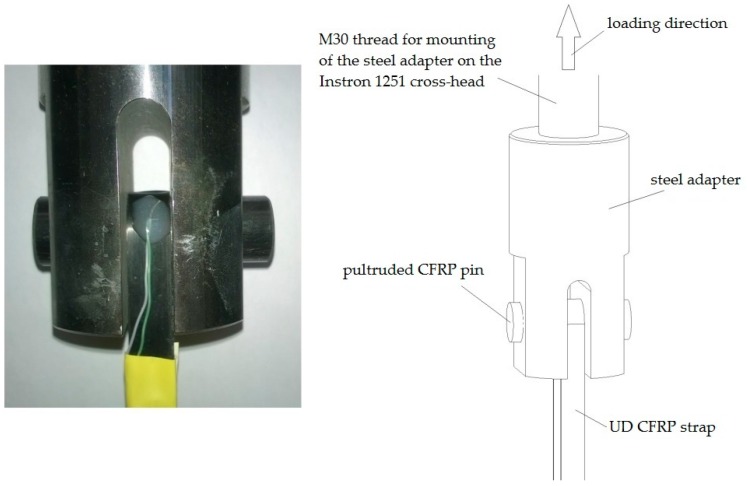
Mounting of the pins and straps in the testing machine.

The ultimate tensile strength (σ_UTS_) and fretting fatigue tests on the straps were conducted on a servo-hydraulic test machine (type 1251, Instron^®^, Norwood, MA, USA). The tensile strength tests were performed under displacement-control at a cross-head speed of 2 mm/min and the fibre parallel strains were measured with a linear encoder and strain gauges with a grid length of 3 mm. The fatigue tests were performed under load-control at a frequency of 10 Hz and a load ratio *R* of 0.1. The 10 Hz frequency was chosen because it represents a realistic loading frequency for e.g., a suspender in a half-through arch bridge repeatedly loaded by lorry axle loads [20] or for a sailing boat rigging used for the anchorage of a mast [21]. This frequency value was also chosen by others [4,5] for the investigation of the fretting fatigue behaviour of CF-EP laminates for mechanical engineering applications. Figure 3 illustrates the mounting of the strap and pin in the testing machine. The pultruded CFRP pin is placed in a fork-like steel adapter that is screwed to the cross-head of the testing machine. The lateral steel surface in contact with the CFRP strap has a surface roughness of Ra ~1 μm. The picture also shows the type K thermocouple that was glued to the strap in order to monitor the temperature development on the outside of the strap in the critical (vertex) area. This was felt to be an important effect to assess. Even though Barron *et al.* [22] found that fibre-dominated orientations exert smaller frequency effects on their fatigue behaviour than matrix-dominated orientations, higher test frequencies lower the dynamic properties of a composite structure due to hysteretic heating [22] and hence lead to more conservative results. Whenever narrow straps were tested, thin copper washers were placed on both sides of the strap to ensure the lateral support of the strap, which is reported to reduce stress concentrations [23].

## 4. Experimental Results and Discussion

### 4.1. Quasi-Static Behaviour

Before the fretting fatigue tests were conducted, five to eight straps were tested for their ultimate tensile strength. The results of the quasi-static tensile tests performed on the wide (12 mm) and narrow (9.7 mm) straps are shown in Table 1. Two different types of straps were tested since the 12 mm wide straps required a lateral machining of the straps in order to fit into the adapter. The 9.7 mm straps, on the contrary, did not require this kind of invasive post-processing which was suspected to negatively influence the mechanical performance of the straps. As can be seen from Table 1, the narrow straps showed a slightly higher σ_UTS_. The elastic modulus on the other hand was slightly below that of the wide straps. Since the results for *E*_11_ and σ_UTS_ of the wide and narrow straps are within the larger of the two respective standard deviations, an influence of the lateral machining on the strap on *E*_11_ or σ_UTS_ was not found. However, all measurements on the narrow straps showed a significantly lower standard deviation, which is tentatively attributed to the machining, inducing larger scatter due to the respective defects.

### 4.2. Fretting Fatigue

**Figure 4:**
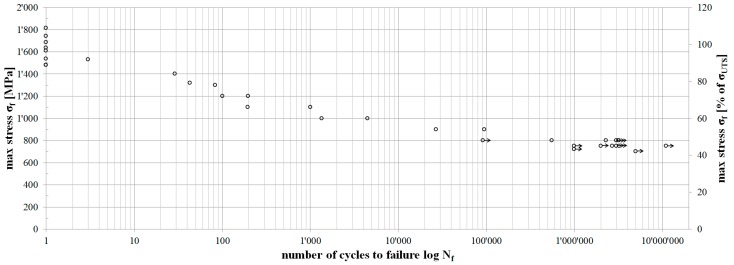
S–N curve of the 12 mm wide straps, listing the stress at failure (σ_f_) as a function of endured load cycles at failure (*N*_f_). Arrows are attached to data points of straps tested without failure, indicating that further load cycles could have been endured.

Figure 4 shows the S–N curve obtained from the fretting fatigue testing of 12 mm wide pin-loaded straps. Eight straps were tested quasi-statically, two straps were tested at each of the six load levels between the quasi-static tests and the fatigue limit and 14 straps were tested at or around the fatigue limit stress. A strap was defined to have reached the fatigue limit once it endured more than 3 million load cycles. Straps tested without failure have an arrow attached to their data point in Figure 4, indicating that further load cycles could have been endured. In order to confirm this, one strap was tested for 11,089,000 load cycles at the fatigue limit stress (750 MPa) without failure.

The residual mechanical properties of straps tested without failure after 1, 2, 3 and 11 million load cycles around the fatigue limit stress were tested in quasi-static tensile tests until failure. The test setup was the same as for the virgin straps. Table 2 lists the results of these measurements.

**Figure 5:**
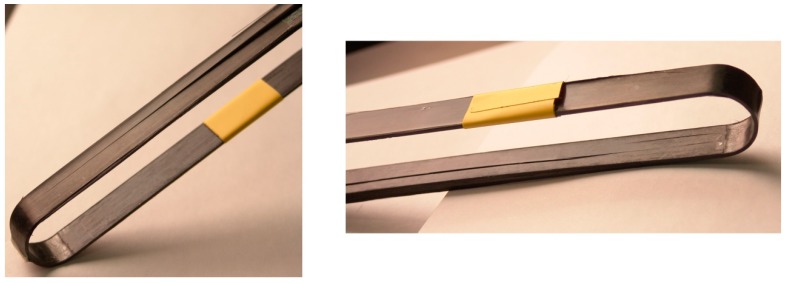
Delamination and longitudinal cracking on a fretting fatigue tested strap. Strap tested for *N* = 9 × 10^4^ cycles at 800 MPa.

**Figure 6:**
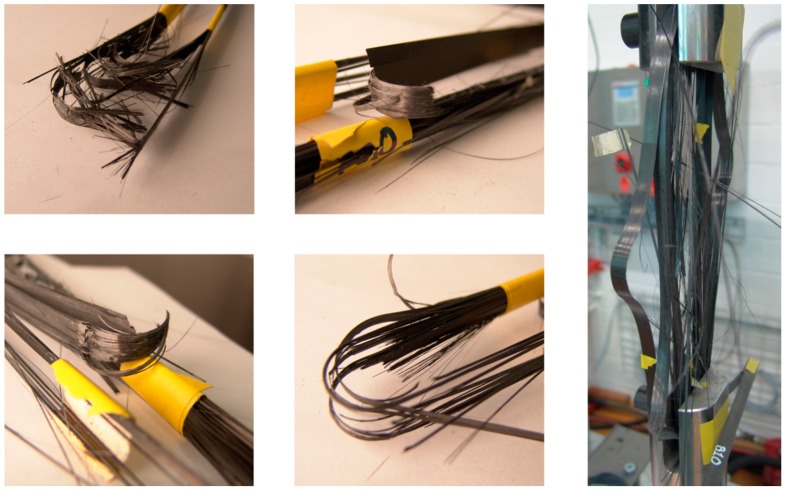
Damage modes in different fretting fatigue loaded straps.

Comparing the residual stiffness’s of straps tested for *N* ≥ 1 × 10^6^ to the stiffness of an average virgin strap reveals a slight stiffness reduction over time of up to 10%. The ultimate tensile strength of the straps on the other hand is hardly affected by the fretting fatigue loading. Furthermore, the standard deviation is much lower in the fatigue tested straps and first signs of (further) damage occur only after the straps have reached ~80% of their ultimate tensile strength. In virgin straps, first signs of damage were observed just above 50% of their ultimate tensile strength. The damage modes of all fretting fatigue tested CFRP straps were similar. First, visible damage always occurred in the form of delamination of the inner- and outermost layers in the shaft with the overlapping plies. The delaminations started at the free ends of the tape and propagated along the shaft until they stopped in the vertex area, see Figure 5. Another damage mode was longitudinal matrix cracking. This damage mode also occurred solely in the inner- and outermost plies and did not propagate over the vertex area of the strap either (Figure 5). However, the failure of all straps was initiated in the vertex areas where clear signs of broken fibres were present (see Figure 6) leading to fibre bursting in one or both shafts. In some straps, the bursting of the innermost plies was accompanied by delamination of the outermost ply (Figure 6, right). This additional delamination predominantly occurred in quasi-static loading until failure of straps that were previously fatigued around the fatigue limit, but was also observed in straps tested at much higher stress levels. Hence, no clear evidence for the correlation between damage mode and stress level was found.

**Figure 7:**
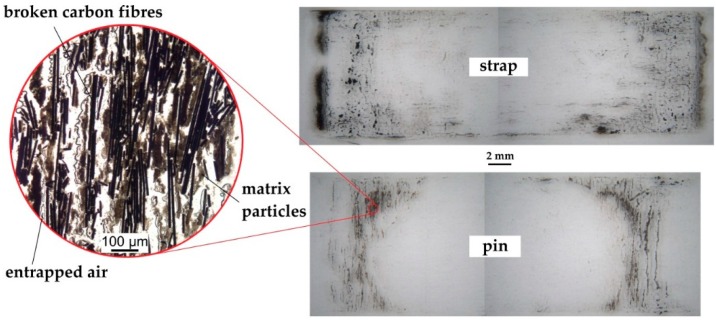
Fretting products of a strap after 3 × 10^4^ load cycles (**top**) and a pin after *N* = 10^6^ (**bottom**). Both images show fretting products from tests conducted at a load level of 720 MPa. The different wear mechanisms on a strap and pin are clearly visible.

The fretting behaviour of the pin-strap contact surface was investigated by placing a transparent adhesive tape on both surfaces just after testing. After pulling the tape off the surfaces, most of the fretting products remained on the tape. These tapes were placed on a sheet of paper and investigated under an optical microscope (ZEISS Axioplan in reflected-light mode). Figure 7 shows two representative pictures from a strap and a pin. Clearly visible are the carbon fibre particle accumulations just outside the vertex area of the strap. In accordance with the literature [4,24,25,26] the main damage mode observed on the strap (sliding of the contacting surface in fibre direction) was fibre thinning, resulting in small carbon particles. The fretting products on the pin on the other hand consist mostly of short, broken and pulled-out carbon fibres with parts of neat resin still attached to the fibres. This is also reported to be typical for sliding perpendicular to the fibres [24,25,26,27,28]. Clear signs of a homogeneous graphite particle film covering the contact area that might act as a lubricant were not detected. However, the graphite particle aggregations just outside the vertex areas of the strap suggest a particle transport from the fretting areas to the free surfaces of the shaft. This would require that the particles do build, at some point, an intermediate film between the pin and the strap which would contribute to a reduction of the coefficient of friction.

**Figure 8:**
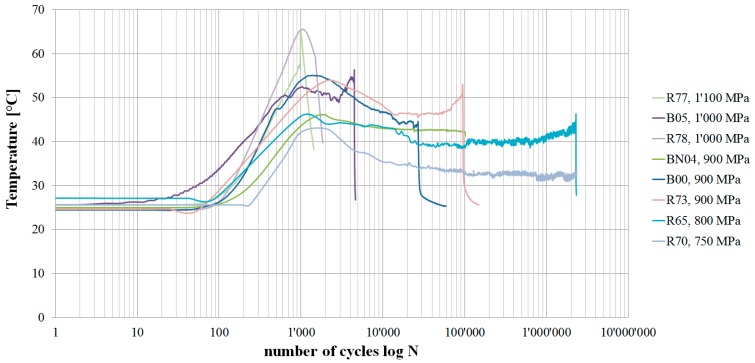
Temperature curves of fretting fatigue tested CFRP straps. Curves shown for straps tested at an upper load level between 750 and 1,100 MPa.

The temperature measurements on the outside of the straps given in Figure 8 show a significant initial temperature increase with a peak within the first 10^4^ load cycles. After this peak, the temperature decreases and levels out before increasing again prior to failure of the strap. This behaviour suggests a running-in process where the contacting surfaces are smoothened. According to the datasheet of the matrix, the glass transition temperature (*T*_g_) of the matrix is between 140 and 145 °C if cured for 1 h at 120 °C followed by 2 h at 140 °C [29]. Since the laminate tested in this study was cured at 140 °C for 2.5 h, the *T*_g_ can be assumed to be in the same temperature range. Hence, the measured maximum temperatures around 65 °C can be considered non-critical.

## 5. Numerical Modelling

The quasi-static tensile tests of the pin-loaded straps resulted in an average σ_UTS_ of 1,624 ± 121 MPa. However, the laminate itself has a σ_UTS_ of 2,567 ± 58 MPa. The analytical model presented in [23] predicts a fibre parallel stress concentration in the vertex area of pin-loaded straps of only 1.1 for the given geometry, lateral support and material properties. In order to investigate this mismatch, a FEA of the pin-strap contact problem under quasi–static tensile loading was conducted in the commercial FEA Software Abaqus/Standard 6.14 (Dassault Systèmes Simulia Corp., Providence, RI, USA) [30]. The local stress distributions in the strap were investigated and the corresponding strains were compared to the experimental results. 

### 5.1. Finite Element Analysis

**Figure 9:**
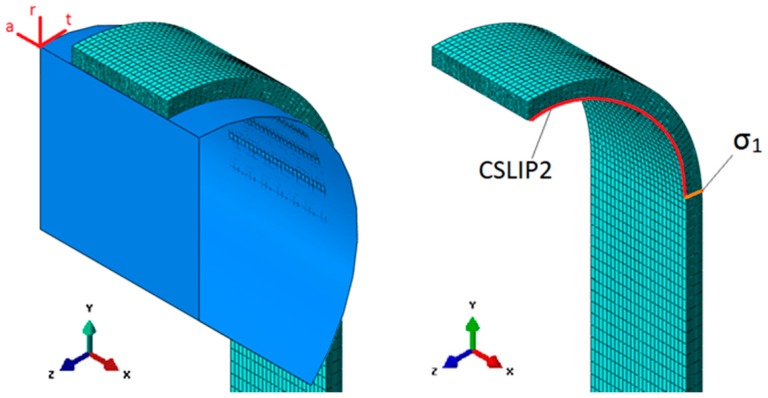
1/8-model of the pin-strap contact problem used for the FEA. The picture on the right illustrates where the measurements of the relative tangential slip (CSLIP2) between the pin and the strap in the fibre direction as well as the local stresses in the fibre direction (σ_1_) were taken.

With the strap’s three symmetry planes, the model used for the FEA could be simplified with a 1/8 model of the strap. The pin was modelled as an analytical rigid surface in order to reduce computational costs without losing the results’ accuracy compared to a solid, deformable CFRP pin. The 1 mm thick and 6 mm wide strap was meshed with 42,028 linear hexahedral elements of type C3D8, resulting in a total of 47,775 nodes. The mesh was locally refined with an approximate element size of 0.1 mm × 0.25 mm × 0.5 mm in the vertex area and 0.1 mm × 0.25 mm × 3 mm in the centre of the shaft. This was found to be a good tradeoff between result accuracy and computational cost. Table 3 lists the elastic material properties that where defined by the engineering constants. For better results, the strap was loaded by defining a pin displacement rather than applying a load to it. The pin-strap interaction was defined over a surface-to-surface interaction, with the pin acting as master and the strap as slave. The coefficient of friction (*cof*) in the contact area varied between *cof* = 0–0.5, which is in the range of values reported in the literature (*cof* = 0.2–0.68, depending on contact material and fibre orientation) [26,27,28]. Figure 9 illustrates the 1/8 model of the strap and shows where the numerical values of the stresses and displacements were read.

### 5.2. FEA Results

**Figure 10:**
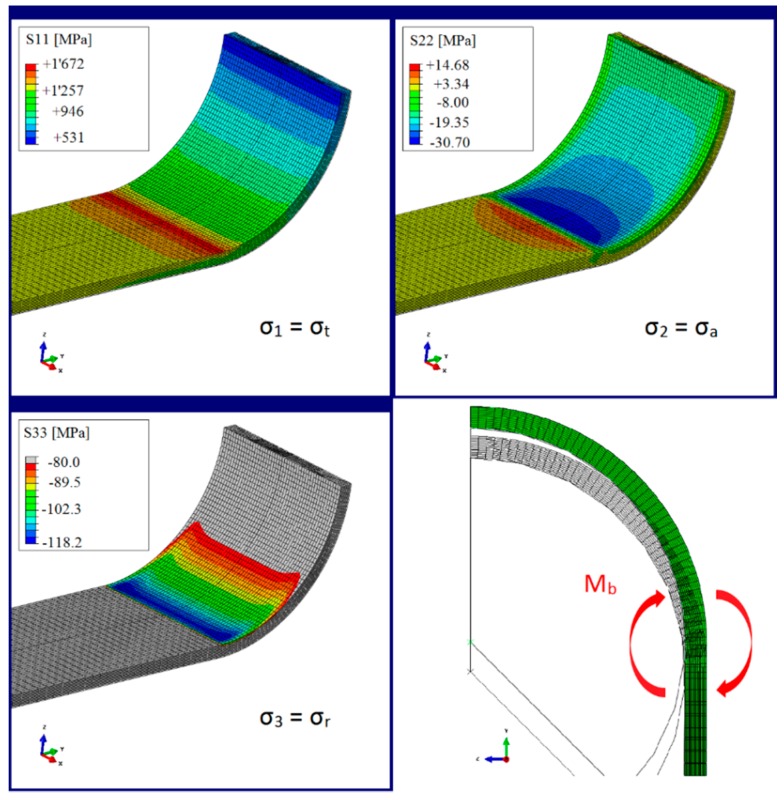
Stress distribution in the vertex area of a 12 mm wide and 1 mm thick strap in contact with an analytical rigid pin (*cof* = 0.5).

With the help of the FEA, the influence of the *cof*, the upper tensile load, the pin diameter and the strap thickness on the stress distribution in the strap and the relative slip between pin and strap could be investigated. The pictures in Figure 10 show the resulting stresses in the vertex area of a 12 mm wide and 1 mm thick strap with an inner radius of 10 mm that was subjected to a pin displacement of 1.05 mm (1,250 MPa shaft-stress). The displacement of the pin causes a bending moment in the vertex area of the strap (bottom right picture) and the resultant significant stress concentrations in fibre direction (σ_1_ = σ_t_) become clearly visible in the top left picture. Furthermore, the profile of the normal stresses in the strap (σ_3_ = σ_r_, bottom left) is similar to the wear pattern observed under the microscope, shown in Figure 7.

The obtained results of an increasing stress concentration of σ_1_ with decreasing inner radius and/or increasing strap thickness are consistent with the literature [23]. However, with the help of the FEA it could be shown how the analytical model presented in [23] underestimates the stress concentrations in the innermost plies. This is due to the fact that it does not consider secondary effects such as the bending moment in the vertex area.

### 5.3. Validity of the FEA

**Figure 11:**
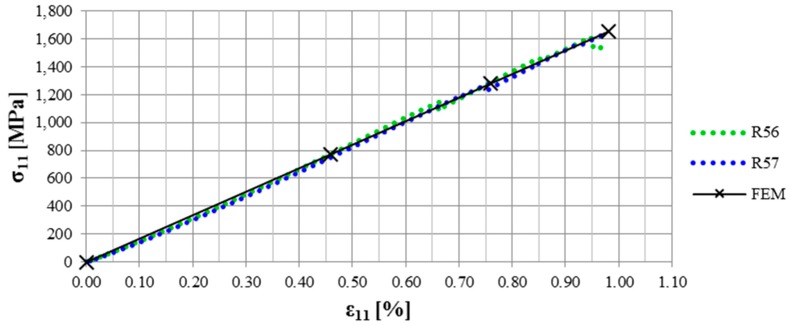
Verification of the FEA. The nodal stresses and strains in the centre of the shaft were taken from the FEA and compared to the experimental results. The fibre parallel shaft strains were measured with strain gauges.

The validity of the FEA is supported by comparing the stress–strain (σ–ε) curves of the straps tested for their ultimate tensile strength to the results obtained from the FEA. The nodal stresses and strains in the FEA were taken at different load (stress) levels and the resulting curve is shown in Figure 11, together with the σ–ε curves from the experiments. It is clearly visible that with a *cof* of 0.5, a good correlation between the numerical model and the experiments was achieved. With a *cof* of 0.5, the stress concentration factor in the vertex area of the strap was around 1.3. This factor alone cannot explain the decrease in σ_UTS_ in the straps entirely. Fibre misalignment, internal stresses and lateral machining of the straps in the vertex area further contribute to the decrease in σ_UTS_. The influence of fibre misalignment and internal stresses on the σ_UTS_ was not investigated in this study. 

## 6. Summary

The fretting fatigue behaviour of pin-loaded CFRP straps under tensile loading was presented in this study. The investigated straps are models for rigging systems used in sailing yachts, pendent cables used in crawler cranes and for suspenders used for half-through arch bridges and have been subjected to ultimate tensile strength and fretting fatigue tests up to 11 × 10^6^ load cycles. All straps were hand-laminated by winding unidirectional, OOA prepreg tapes around an aluminium mould and compressing the laminate between two aluminium clamps. Ultimate tensile strength tests of the laminate were performed to determine the laminate’s mechanical properties needed for the FEA. Eight 12 mm and five 9.7 mm wide straps were tested in quasi-static tensile loading until failure and the results were compared to coupon tests and FEA. A total number of 26 straps, all 12 mm wide, were subjected to fretting fatigue loading at a load ratio *R* of 0.1 and a frequency *f* of 10 Hz and the results were used to generate an S–N curve. The following conclusions can be drawn on the basis of the study described in this paper:

1. The pin-loaded CFRP straps showed a fatigue limit stress of 750 MPa, which corresponds to approximately 46% of their ultimate tensile strength. The fatigue limit was defined to be reached once a strap endured more than 3 × 10^6^ load cycles, and one strap was tested for 11.09 × 10^6^ load cycles to confirm this.
2. The experimentally determined fatigue limit corresponds to the matrix fatigue limit strain of 0.6%, which is reported in the literature [16] to be the ultimate lower bound for a UD fibre reinforced composite fatigue limit.
3. The ultimate tensile strength of the straps is significantly reduced by stress concentrations in the vertex area. In the investigated case of a 1 mm thick strap with an inner radius of 10 mm, the fibre parallel stress concentration factor in the innermost plies of the strap was 1.3.
4. The stress concentrations in the vertex area of the strap depend on the coefficient of friction, the applied load and the pin and strap geometry, as was shown in previous studies [23].
5. The influence of the lateral machining of the straps on their stiffness and ultimate tensile strength could not be shown. However, measurements on straps that were laterally machined showed a higher standard deviation of stiffness and strength.
6. Clear signs of a graphite film acting as lubricant between pin and strap were not found. However, the carbon fibre particle accumulations just outside the vertex area of the inner strap surface require a transport of these particles from the fretting areas to the outside. This in turn means that a particle film is present at some point and contributes to a reduction of the coefficient of friction.

## Figures and Tables

**Table 1:** Quasi-static tensile strength and stiffness of wide (left) and narrow (right) straps. The fibre parallel elastic modulus *E*_11_ [GPa] was calculated following [17] and was measured with strain gauges (*E*_11_SG_) and a linear encoder (*E*_11_LE_). The composite strains at failure ε_cf_ [%] were measured with the strain gauges. Wide straps are labelled with B, R or T, depending on the author who manufactured them, followed by the number of the strap. Narrow straps are labelled with BN and the number of the strap.

Strap	σ_UTS_ [MPa]	*E*_11_SG_ [GPa]	*E*_11_LE_ [GPa]	ε_cf_ [%]	Strap	σ_UTS_ [MPa]	*E*_11_SG_ [GPa]	*E*_11_LE_ [GPa]	ε_cf_ [%]
Average	1,624 ± 121	177.8 ± 8.6	175.8 ± 12.1	0.99 ± 0.04	Average	1,714 ± 55	174.4 ± 1.1	166.2 ± 6.2	1.00 ± 0.05
B31	1,741	186.3	177.0	1.04	BN06	1,688	-	162.6	-
B32	1,538	187.7	180.3	0.95	BN09	1,663	-	158.7	-
B33	1,482	169.3	158.9	1.01	BN11	1,781	174.4	168.0	1.04
R56	1,611	174.3	-	1.00	BN15	1,767	173.4	166.4	1.02
R57	1,686	171.6	-	0.97	BN20	1,673	175.5	175.2	0.95
B01	1,637	-	187.2	-					
B14	1,480	-	-	-					
T72	1,815	-	-	-					

**Table 2:** Straps tested for their residual strength and stiffness properties after 1, 2, 3 and 11 million load cycles at an upper stress level (σ_u_) of 720–750 MPa. The fibre parallel elastic modulus *E*_11_ was calculated from linear encoder measurements (*E*_11_LE_). Straps are labelled with B or R, depending on the author who manufactured them, followed by the number of the strap.

Strap	σ_u_ [MPa]	Number of cycles tested	σ_UTS_ [MPa]	σ_UTS_ [%]	*E*_11_LE_ [GPa]	*E*_11_LE_ [%]
Average	-	-	1,620 ± 65	100.0	165.9 ± 4.0	100.0
B11	750	1.00 × 10^6^	1,575	97.2	170.2	102.6
B12	720	1.00 × 10^6^	1,627	100.4	166.6	100.5
B13	750	2.00 × 10^6^	1,618	99.9	-	-
B15	750	3.00 × 10^6^	1,725	106.5	166.0	100.1
R70	750	11.09 × 10^6^	1,557	96.1	160.6	96.8

**Table 3:** Engineering constants used for the FEA.

Material	*E*_11_ [MPa]	*E*_22_ [MPa]	*E*_33_ [MPa]	ν_12_ [-]	ν_13_ [-]	ν_23_ [-]	*G*_12_ [MPa]	*G*_13_ [MPa]	*G*_23_ [MPa]
IMS60	168,000	8,000	8,000	0.27	0.27	0.39	4,600	4,600	3,200
